# Something of Interest

**Published:** 1888-06

**Authors:** 


### SOMETHING OF INTEREST.

In this number is published the introduction to a series of articles by Dr. Miller, upon "Pathogenic Bacteria of the Human Mouth," which, if we may judge by the matter already in hand, will fully equal in value and interest anything that has yet been published from his pen. The drawings that we have received indicate—what had already been established—that he is quite as happy with the pencil as with the pen, and if we succeed in having them faithfully reproduced they will be as valuable to every student of scientific dentistry as is the text. Prof. Miller now stands first in his chosen field, and when he speaks it is as one having authority, for the thorough and exhaustive studies and observations in which he has for years been engaged, a protracted series of connected experiments extending over long periods of time, all conducted in accordance with the most rigid scientific law, enable him to draw definite and positive conclusions when such are possible. There is probably no one now living who is so thoroughly versed in oral bacteriology as is Prof. Miller, and the results of his exhaustive research will be laid before the readers of THE INDEPENDENT PRACTITIONER in the series of articles now just commenced. We need not ask their careful reading, for no dentist who makes any pretense to even the most superficial knowledge of the subject can avoid their study.

When the results of the observations of Pasteur, Koch and others in bacteriology were first published, and the attention of the medical men and scientists of the world was absorbed in the new revelations, two schools of pathologists sprung up, the first claiming, with the renowned observers, that the micro-organisms were the sources of contagion and the direct cause of the diseases to which they were peculiar, and the second that they were but the accidental occupants of the products of diseased action, which were their natural habitat; that they were scavengers, proliferating within and rendering innocuous septic matter. The debate between the adherents of the two theories was sometimes determined and bitter, the arguments on each side being met with rebutting testimony, and experiment encountering experiment. It seemed extremely difficult to positively demonstrate beyond the reach of cavil the truth of either theory. But when Prof. Miller, by means of pure cultivations of the *delta* organism (Miller), artificially produced true caries of the human tooth, as first published in THE INDEPENDENT PRACTITIONER in 1884, a fact was established which there was no gainsaying, and the opponents of the theory of the pathogenic character of certain bacteria were effectually silenced.

The man who did this, who artificially produced such definitive changes in a tissue like that of the human tooth when removed from all vital connection, may well be allowed to speak authoritatively on oral pathology, and those who have any respect for earnest, painstaking, scientific investigation, will listen respectfully to what he has to say, for he has demonstrated his fitness to stand as a teacher.

In this connection, it gives us pleasure to announce that Prof. Miller will visit this country during the coming summer. He will arrive about the middle of August, and will remain for some weeks. But he comes for rest and recuperation—a rest sorely needed—and he will not probably be able to engage in any professional work in this country.

